# The Effect of Three Different Ketogenic Diet Protocols on Migraine and Fatigue in Chronic and High-Frequency Episodic Migraine: A Pilot Study

**DOI:** 10.3390/nu15204334

**Published:** 2023-10-11

**Authors:** Yan Tereshko, Simone Dal Bello, Cherubino Di Lorenzo, Alice Pittino, Francesca Filippi, Enrico Belgrado, Christian Lettieri, Giovanni Merlino, Gian Luigi Gigli, Mariarosaria Valente

**Affiliations:** 1 Clinical Neurology Unit, Udine University Hospital, Piazzale Santa Maria Della Misericordia 15, 33100 Udine, Italyfrancescafilippi.nutrizionista@gmail.com (F.F.); giovanni.merlino@asufc.sanita.fvg.it (G.M.); mariarosaria.valente@uniud.it (M.V.); 2 Department of Medico-Surgical Sciences and Biotechnologies, Sapienza University of Rome Polo Pontino, 04100 Latina, Italy; 3 Neurology Unit, Udine University Hospital, Piazzale Santa Maria Della Misericordia 15, 33100 Udine, Italy; enrico.belgrado@asufc.sanita.fvg.it; 4 Department of Medicine (DAME), University of Udine, Via Colugna 50, 33100 Udine, Italy

**Keywords:** ketogenic diet, fatigue, headache, migraine, LGID, VLCKD, KD

## Abstract

Aims: We aimed to evaluate the efficacy of three different ketogenic diets on migraine and fatigue in chronic and high-frequency episodic migraineurs. Methods: 76 patients with migraine were treated with the KD for at least three months. Three different KD protocols were used (2:1 KD, LGID, and VLCKD). We evaluated the fatigue severity scale (FSS), migraine frequency, migraine intensity, MIDAS, and HIT-6 at the baseline and 3-month follow-up, and we compared the results. We also correlated the mean FSS reduction with the mean migraine frequency, migraine intensity, BMI, fat mass, free-fat mass, MIDAS, and HIT-6 reduction. Results: FSS improved from 4.977 ± 1.779 to 3.911 ± 1.779 at the 3-month follow-up (*p* < 0.001). This improvement was significant in both high-frequency and chronic migraineurs. Moreover, the three KD protocols effectively improved migraine intensity, frequency, MIDAS, and HIT-6. There was a mild correlation between mean FSS reduction (*p* < 0.001), mean MIDAS (*p* = 0.001), and HIT-6 (*p* = 0.002) reduction. Conclusions: The VLCKD, LGID, and 2:1 KD may improve migraine intensity, frequency, and fatigue in chronic and high-frequency episodic migraineurs.

## 1. Introduction

Migraine is a primary episodic headache disorder caused by altered excitability of the central nervous system (CNS) and characterized by various combinations of neurological, gastrointestinal, and autonomic features. It ranks among the world’s most disabling medical illnesses. The economic and societal effect of migraine is substantial: it affects patients’ quality of life and impairs work, social activities, and family life [1].

In addition to headache, typical accompanying symptoms of migraine include nausea and vomiting, photo-, phono-, and/or osmo-phobia. Blurry vision, nasal stuffiness, anorexia, hunger, tenesmus, diarrhea, abdominal cramps, polyuria, facial pallor, sensations of heat or cold, and sweating might occur. Depression, fatigue, anxiety, nervousness, irritability, and concentration impairment are common [1]. Mood, cognition, and fatigue disturbances can occur during migraine but also before and after.

Patients often describe fatigue as a lack of energy, mental or physical tiredness, diminished endurance, and prolonged recovery after physical activity [2]. Fatigue is defined as difficulty sustaining or initiating voluntary activity [3]. The recognition of fatigue in migraineurs in clinical practice is lacking, but it is estimated that up to 60% of migraineurs report significant fatigue [4]. Moreover, fatigue can be one of the migraine triggers, and its presence is associated with a higher frequency of migraine episodes [5,6,7]. Chronic fatigue also has a high economic relevance due to its association with work absence and productivity loss [8,9]. Women migraineurs with comorbid depression or anxiety are likelier to report fatigue symptoms [6]. Fatigue and headache can often coexist in the same subject, not only in migraine patients. Indeed, these two symptoms have been described in different conditions, like long COVID-19 [10] and viral infections in general [11], kidney disease [12], mild traumatic brain injury [13], side effects of vaccination [14], fibromyalgia [15], and premenstrual syndrome [16]. Fatigue is a multifactorial symptom influenced by mood, sleep, overall health, and medications. The ketogenic diet (KD) is a nutritional approach that was developed in neurology for childhood epilepsy treatment [17]. It is based on reducing carbohydrates, an adequate but limited protein intake, and satisfying caloric requirements through lipids [18]. The common goal of all KD diet protocols is to induce the production of ketones as an energy substrate to fulfill the body’s calorie needs. The ketones produced in this diet are highly energetic, and a KD can effectively raise the ATP/ADP ratio in the brain [19]. There are different ketogenic protocols used in clinical practice to treat migraine, and these include the low-glycemic-index diet (LGID) [20,21], the low-calorie ketogenic-diet (LCKD) [22], the very-low-calorie ketogenic-diet (VLCKD) [22], the Modified Atkins Diet (MAD) [23,24], and the Classic KD [22,25]. Classic KD is characterized by a very low carbohydrate intake, increased fats, and an adequate intake of proteins. The LGID is also characterized by a drastic reduction in carbohydrates, regular intake of proteins, and an increase in fats; however, the carbohydrates must have a glycemic index < 50, meaning that they do not significantly increase blood glucose. The VLCKD is a very restrictive ketogenic diet in which the calories derived from fats account for 44% of the overall calories daily, while carbohydrates and proteins contribute less at 43% and 13%, respectively; this diet protocol is mostly used in obese patients. The classical KD and other ketogenic diet protocols have proven effective in treating migraines, and studies regarding their application in the treatment of fatigue are currently underway; however, there are currently no data regarding the efficacy of KDs on fatigue in migraineurs. This study aims to quantify the fatigue of chronic and high-frequency episodic migraineurs before and after diet therapy.

## 2. Methods

### 2.1. Study Design, Participants, and Eligibility

This was a retrospective single-center pilot study with prospectively collected data. We offered diet therapy as a preventive therapy for migraine patients who were interested in alternative therapies and unwilling to start conventional approaches. We collected data from 76 patients with a clinical diagnosis of migraine (both chronic and high-frequency episodic migraine) who underwent a KD as a preventive measure for their migraine from January 2020 to December 2022 in our nutritional outpatient clinic (Clinical Neurology Unit, Ospedale S. Maria della Misericordia, Udine) and fulfilled the requirements. These chronic or high-frequency episodic migraineurs had to be >18 years old and they must have had 3-month headache diaries compiled before the diet initiation and during the follow-up. Exclusion criteria were as follows: the presence of organic or mental diseases aside from major depressive disorder or anxiety disorder, pregnancy and breastfeeding, intellectual impairment, prior surgery, and BMI < 18 Kg/m^2^; the presence of anti-migraine preventive medication was permitted during the follow-up only if it was present at least three months before the initiation of diet therapy; other therapies were not allowed. Symptomatic medication intake was allowed if limited to less than 15 monthly administrations and involved only triptans or NSAIDs; opioid drugs were not allowed. The presence of medication overuse headache before the study was permitted. The data regarding the demographics, migraine features, comorbidities, previous preventive therapies, and headache diary were collected before patients began the diet. We also collected baseline MIDAS, HIT-6, and FSS scores. The diet protocol (2:1 KD, LGID, or VLCKD) was decided based on the patient’s BMI. Our nutritionist assessed the anthropometric data, the body mass index (BMI), the fat mass (FM), and the free-fat mass (FFM); to assess FM and FFM, the Bioelectrical Impedance Analysis (BIA) 101 BIVA PRO (Akern^®^, Pisa, Italy) was used. Adherence to the diet was assessed by our nutritionist one month after the diet initiation. At the end of the three months, we collected the data regarding the MIDAS, HIT-6, FSS, and headache diary, and our nutritionist re-evaluated the anthropometric measures as well as the BMI, the FM, and the FFM.

### 2.2. Ethical Aspects

This study was conducted in accordance with the Declaration of Helsinki. This study was approved by the Institutional Review Board of the University of Udine IRB-DAME (Prot IRB: 103/2022; Tit III cl 13 fasc. 8/2022). All the patients formally consented to nutritional treatment with diet as a preventive therapy for migraine and for their data to be used for research.

### 2.3. Ketogenic Diet Therapy

The 2:1 KD was prescribed to patients with a body mass index that ranged from 18.5 to 24.9 Kg/m^2^, and the total calories ranged from 1600 to 2300 Kcal a day; the low-glycemic-index diet (LGID) was prescribed to those with a body mass index between 25 and 29.9 Kg/m^2^, and the calories ranged from 1300 to 1500 Kcal a day. Finally, the VLCKD was prescribed to patients with a body mass index equal to or over 30 Kg/m^2^ to reduce their weight; in this case, the daily calorie intake ranged from 600 to 800 Kcal. Our nutritionist was counseled to aid the patients in the customization of their diet to achieve higher adherence. In each diet, the content of carbohydrates was fixed at 30 g a day; in the case of the LGID, these carbohydrates had a glycemic index of less than 50. The content of proteins and fats was also fixed and was determined by considering the anthropometric measures, the BIA (fat mass and free-fat mass), and the level of daily physical activity; moreover, the intake of proteins was at least 75 g a day to preserve lean mass, and the maximum intake was 2 g per kg of ideal weight to avoid gastric, renal, and vascular abnormalities. With these diets, we aimed to reduce fat by preserving lean mass. In particular, the protein content was calculated based on the lean mass measured with the BIA, and conversion in grams was achieved considering the ideal weight and the level of daily physical activity. In the case of the LGID, the fats were equal to the sum of carbohydrates and proteins (1:1 ratio), while in the 2:1 KD, the quantity of fats was double the sum of carbohydrates and proteins. In the VLCKD, since the carbohydrates are fixed at 30 g a day (12–20% of daily calories) and the protein intake is at least 75 g a day (37.5–50% of daily calories), the remaining calories delivered by fats account for 30–47.5% of the daily calories; to achieve 600–800 Kcal a day, the ratio was varied from 0.43:1 to 0.9:1. The duration of each diet was at least 3 months and a maximum of 6 months.

### 2.4. Fatigue Severity Scale

Fatigue is a symptom and is measured via self-reporting. The fatigue severity scale (FSS) is a 9-item scale that measures the average amount of fatigue experienced by the patients during the previous days (Table 1). It measures the level of fatigue and its effect on a person’s activities and lifestyle in patients with various disorders. It was initially designed for multiple sclerosis and systemic lupus erythematosus; however, it has also been used in migraine [4]. It measures how much fatigue, in chronic disorder settings, limits patients’ cognitive and physical functioning. The patient rates the severity of their fatigue symptoms for each item; a low value indicates disagreement, while a higher value indicates agreement. In particular, each statement is scored on a 7-point scale, from 1 (strongly disagree) to 7 (strongly agree). The results are added, and the sum is divided by 9; it is essential to point out that some authors use the total score instead. A minimum score of 4 (or 36) is needed to report the presence of pathologic fatigue [26].

### 2.5. MIDAS and HIT-6 Scales

The Migraine Disability Questionnaire (MIDAS) is a self-report questionnaire that assesses the level of disability due to migraine in the previous three months [27]. It has seven items, and the first five items consider the number of missed or reduced-productivity days due to migraine in different aspects of daily living (schoolwork or work, housework, family, social, and leisure activities); the last two items investigate the intensity and the frequency of migraine in the previous three months. Only the sum of the first five items contributes to the total score; the patients are then placed in one of the four categories of disability: little or no disability (0–5), mild disability (6–10), moderate disability (11–20), or severe disability (>20).

The Headache Impact Test (HIT-6) is also a self-report questionnaire designed to assess migraine’s impact on quality of life (pain, social functioning, psychological stress, cognitive function) [28]. Three items are related to the previous four weeks; the remaining three items have no specific period specified. The patient quantifies each item on a frequency scale from 1 to 5 (never, rarely, sometimes, very often, and always), and the score for each response is quantified as 6, 8, 19, 11, and 13 accordingly. The sum of each item determines the total score; the total score ranges from 36 to 78. The patient is then categorized into one of the four levels of severity: little or no impact (<50), some impact (50–55), substantial implications (56–59), and very severe impact (>59).

### 2.6. Primary Endpoint

We aimed to assess the effect of 3 months of KD therapy in chronic and high-frequency episodic migraineurs on fatigue utilizing the fatigue severity scale (FSS). Moreover, we evaluated the effect on migraine frequency (days per month), intensity, MIDAS, and HIT-6.

### 2.7. Secondary Endpoint

To assess the correlation between the mean reduction in FSS and the mean reduction in HIT-6, MIDAS, FM, FFM, migraine frequency and intensity, and BMI.

### 2.8. Data and Statistical Analysis

Since this was a pilot study, the power of the study was not calculated; a descriptive analysis of the study population’s main features was performed using mean ± SD for continuous variables and absolute and relative frequencies for categorical variables. A Shapiro–Wilk test was used to assess the normal distribution of data. Group comparisons were performed as appropriate using a *t*-test or Mann–Whitney’s test. A paired *t*-test or a Wilcoxon test was used to compare the clinical data at the baseline and after three months of diet. Correlation analysis was performed with Spearman’s test. All analyses used Stata/SE (version 15.1, StataCorp, College Station, TX, USA) for Mac OS. All 2-tailed statistical significance levels were set at *p* < 0.05.

## 3. Results

Seventy subjects with migraine were treated with diet for three months; our population mostly comprised women (83.86%). The mean age of our study sample was 45.895 ± 14.773 years. Forty-five patients (59.21%) had chronic migraine, while thirty-one (40.79%) had high-frequency episodic migraine. The mean migraine duration was 22.461 ± 17.364 years, and the number of previous prophylaxes for migraine was 2.474 ± 2.069. Fourteen subjects underwent the VLCKD treatment (twelve chronic migraineurs), twenty-one the 2:1 KD diet (nine chronic migraineurs), and the remaining forty-one patients the LGID protocol (twenty-three chronic migraineurs). Forty-two patients were on concomitant prophylaxis for at least three months before the start of the diet (see Table 2 for details on the sample’s demographics).

Fifty subjects (65.79%) had an FSS score of 4 or higher. Twenty-nine patients out of forty-five with chronic migraine had pathologic fatigue (64.44%); this ratio was similar in the high-frequency episodic migraineurs, with twenty-one patients having pathologic fatigue (67.74%).

The diet was able to reduce both the frequency (18.197 ± 8.408 vs. 8.750 ± 9.436; *p* < 0.001) of migraine days and the intensity (8.145 ± 1.055 vs. 5.355 ± 2.779; *p* < 0.001) of the migraine attacks. There was also a reduction in the MIDAS score (76.079 ± 74.257 vs. 37.079 ± 59.076; *p* < 0.001) and HIT-6 score (65.092 ± 6.575 vs. 54.105 ± 13.241; *p* < 0.001). The diet reduced the fat mass and the BMI by preserving the free-fat mass (see Table 3 for details).

KD treatment improved the fatigue symptom; every item in the fatigue severity score improved significantly, reducing the total score from 4.977 ± 1.644 to 3.911 ± 1.779 (*p* < 0.001), as shown in Table 4. In particular, both chronic and high-frequency episodic migraineurs had pathologic fatigue at the baseline; after three months of diet, the mean score was significantly reduced to a normal value. Moreover, the three diets were able to improve fatigue significantly.

**Figure 1:**
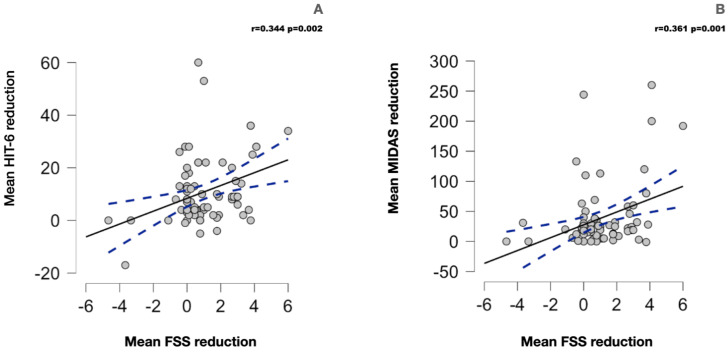
(**A**) The scatterplot for the correlation between the mean reduction in the FSS and the mean reduction in HIT-6 score. (**B**) The correlation between the FSS score’s mean reduction and the MIDAS score’s mean reduction. The blue dotted line represents the 95% confidence interval.

Spearman’s analysis detected a mild positive correlation (see Figure 1) between the mean reduction in the FSS and the mean reduction in MIDAS (r = 0.361; *p* = 0.002) and HIT-6 (r = 0.344; *p* = 0.001) scores but not with the mean reduction in the frequency and the intensity of migraine or with the mean reduction in the fat mass or the BMI (see Table 5 for the detailed statistics).

The side effects were reported as mild. A total of 12 patients reported mild constipation (5 2:1 KD patients, 5 LGID patients, 2 VLCKD patients), 2 patients reported diarrhea (1 VLCKD patient, 1 LGID patient), 19 patients had abdominal pain (10 LGID patients, 5 2:1 KD patients, 4 VLCKD patients), and 2 LGID patients had occasional nausea. Blood tests after the 3 months of diet did not show hyperlipidemia or hyperuricemia. 

## 4. Discussion

In our study, the KD effectively reduced fatigue levels as measured using the FSS in migraine patients, from mean pathological values at baseline of 4977 ± 1644 to values of 3911 ± 1779 3 months after starting the diet. This significance remained when the sample was analyzed considering chronic and high-frequency episodic migraineurs. Moreover, all three diets were able to reduce fatigue significantly. Migraine frequency and intensity also improved significantly (see Table 3).

Furthermore, we observed that the mean reduction in the FSS correlates with the mean reduction in the HIT-6 (*p* = 0.002) and MIDAS (*p* = 0.001) scores; this correlation was mild and indicates a correlation between the reduction in fatigue and migraine disability. This could be related to the effect of the ketogenic diet on both disorders. However, fatigue impacts migraine disability and quality of life [29]; it is possible that the reduction in fatigue could have had some impact on migraine disability and quality of life, but the test evaluated only correlation and not causation. Moreover, it is likely that the significant reduction in migraine frequency, intensity, and migraine disability could have decreased fatigue in our sample; moreover, our data regarding the KD’s effect on migraine are supported by the existing literature [21,23,30]. In contrast, the mean reduction in migraine frequency, intensity, FM, and BMI do not correlate with the mean reduction in FSS. 

The prevalence of the symptom fatigue in migraineurs is high and reported in about 58.8% of patients [4]. The reason for this high prevalence is likely the pathogenetic mechanisms underlying migraine and fatigue. Indeed, the etiologic mechanisms underlying fatigue still need to be better understood. Still, dysfunctions in the mitochondrial structure, mitochondrial function (mitochondrial enzymes and oxidative/nitrosative stress), and mitochondrial energy metabolism (ATP production and fatty acid metabolism), together with immune response and genetics, were investigated as potential contributors to fatigue [2]. Likewise, a significant role of mitochondria in the pathophysiology of migraine is also recognized [31], and mitochondrial-targeted treatments improve migraine in a pharmacogenetic fashion [32]. Ketones are a mitochondrial booster [33], increasing ATP production. Thus, the underlying rationale for using the ketogenic diet to treat fatigue in migraine is to improve mitochondrial dysfunction, which may be a common cause of both disorders. The KD may improve fatigue by improving sleep quality [34]; fatigue and sleep disorders are frequently associated [35], and the KD stimulates the synthesis of glutamine and thus GABA, modulating sleep by increasing the slow-wave activity of NREM sleep [36]. Moreover, fatigue correlates to low serum corticotropin and low serum cortisol, which induces an increase in pro-inflammatory cytokines; the KD can reduce pro-inflammatory cytokines and thus fatigue [3]. Fatigue is influenced by motivational input and feedback from the motor, sensory, and cognitive systems, which contribute to establishing the level of perceived effort [3]; the effect of the KD on reducing migraine intensity and frequency may improve the effort perception in task performance. 

Indeed, the KD has been shown to reduce the frequency and intensity of migraine and improve fatigue in patients with multiple sclerosis [25]. 

In a recent study on 226 migraineurs, 58.8% had pathologic fatigue [4]. However, it is important to note that the cut-off to define pathologic fatigue with the FSS was 3.22, not 4. In this study, the FSS score was significantly associated with age, age of onset, the visual analog scale (VAS) depicting headache intensity, photophobia, phonophobia, and the scores of the ASC-12, the MIDAS, the ESS, the ISI, the PHQ-9, and the GAD-7. The strongest predictor for the FSS was the PHQ-9 (*p* < 0.001), followed by age (*p* = 0.002), the ISI (*p* = 0.016), and the VAS (*p* = 0.018); moreover, there was an inverse correlation between the FSS score and three-dimensional scores of the MSQ (*p* < 0.001) [4]. 

In another study, the ratio of migraineurs with fatigue was higher in those with a higher frequency, ranging from 33.8% in those with migraines 1–4 days per month to 39.1% in those with migraines 8 or more days per month; moreover, the control group had lower fatigue than migraineurs (23.7%), but the difference was not statistically significant [37]. In an Italian study in which 100 migraineurs were compared to 100 healthy controls, the control group had significantly lower fatigue than the migraine group (5% vs. 62%) [38]; however, the cut-off for the FSS used was 3 instead of 4. A Brazilian study with 62 chronic migraine patients showed that 84.1% had pathologic fatigue; the cut-off for the FSS was 3 (27) instead of 4 [6]. As pointed out, there is heterogeneity in the cut-off used for the FSS, meaning that there is difficulty in estimating the real frequency of fatigue in the migraine setting.

Snetselaar et al. [39] conducted a systematic review examining 12 randomized trials evaluating the effectiveness of different types of diets in patients with multiple sclerosis. Eight dietary interventions were compared: low-fat, Mediterranean, ketogenic, anti-inflammatory, paleolithic, fasting, calorie restriction, and control (usual diet). Paleolithic, low-fat, and Mediterranean diets showed greater reductions in fatigue than the control [39]. Considering these results, in the future, we could compare the effectiveness of the KD on curbing fatigue in migraine patients compared with other dietary regimens.

### Limitations of the Study

Our study has some limitations. First of all, the sample size was relatively small, and larger studies are needed to confirm our results. Moreover, the uncontrolled retrospective design of this study is another limitation. Ketonuria and/or ketonemia were not assessed since they are recommended in case of scarce results or no response to the therapy; moreover, in our study, the three diet protocols were meant to induce a mild level of ketosis due to low ketone body production, and, therefore, the detection was not relevant. We cannot exclude a placebo effect since this was not a double-blinded study. Our sample comprised patients interested in following a different approach to migraine prevention; therefore, they may not represent the whole population of migraineurs.

## 5. Conclusions

Pathologic fatigue is an important symptom that migraine patients frequently report. The VLCKD, LGID, and 2:1 KD improved migraine frequency, migraine intensity, and fatigue in chronic and high-frequency episodic migraineurs. The effect on fatigue could be related to decreased migraine disability, frequency, and intensity. Moreover, a mild correlation exists between the mean reduction in FSS and the MIDAS and HIT-6 scores. More studies, with larger samples and a double-blinded placebo-control design, are needed to confirm our results.

## Figures and Tables

**Table 1:** The items of the FSS.

During the Past Week, I Have Found That	Disagree ←→ Agree
1. My motivation is lower when I am fatigued	1 2 3 4 5 6 7
2. Exercise brings on my fatigue	1 2 3 4 5 6 7
3. I am easily fatigued	1 2 3 4 5 6 7
4. Fatigue interferes with my physical functioning	1 2 3 4 5 6 7
5. Fatigue causes frequent problems for me	1 2 3 4 5 6 7
6. My fatigue prevents sustained physical functioning	1 2 3 4 5 6 7
7. Fatigue interferes with carrying out certain duties and responsibilities	1 2 3 4 5 6 7
8. Fatigue is among my three most disabling symptoms	1 2 3 4 5 6 7
9. Fatigue interferes with my work, family, or social life	1 2 3 4 5 6 7

**Table 2:** The demographics of our sample.

Variable	Mean ± SD or N° (%)
Number of patients	76
Age	45.895 ± 14.773
Female sex	58/70 (84.21%)
Smoking	13/76 (17.11%)
Chronic migraine	45/76 (59.21%)
Migraine duration (years)	22.461 ± 17.364
N° previous prophylaxes	2.474 ± 2.069
Concomitant prophylaxis	42/76 (55.26%)
MOH	37/76 (40.79%)
Depression	7/76 (9.21%)

**Table 3:** The data at the baseline and after three months of diet. A *t*-test or a Mann–Whitney test (when the data distribution was not normal) was used to compare data; significance was determined at a *p*-value of 0.05. Legend = FM: fat mass; FFM: fat-free mass; BMI: body mass index; MIDAS: Migraine Disability Assessment Test; HIT-6: Headache Impact Test 6; NRS: Numeric Pain Rating Scale; T1: baseline assessment; T1: after 3 months of diet assessment.

	T0	T1	*p*-Value
MIDAS	76.079 ± 74.257	37.079 ± 59.076	<0.001
HIT-6	65.092 ± 6.575	54.105 ± 13.241	<0.001
Frequency (days/month)	18.197 ± 8.408	8.750 ± 9.436	<0.001
Intensity (NRS)	8.145 ± 1.055	5.355 ± 2.779	<0.001
FM (Kg)	24.262 ± 11.338	18.328 ± 10.118	<0.001
FFM (Kg)	49.822 ± 8.565	49.288 ± 8.196	0.136
BMI (Kg/m^2^)	26.715 ± 5.961	24.372 ± 5.044	<0.001

**Table 4:** The items and the global score of the FSS at the baseline and after three months of diet. A *t*-test or a Mann–Whitney test (when the data distribution was not normal) was used to compare data; significance was determined at a *p*-value of 0.05. Legend = FSS: fatigue severity scale; item 1: my motivation is lower when I am fatigued; item 2: my exercise brings on my fatigue; item 3: I am easily fatigued; item 4: fatigue interferes with my physical functioning; item 5: fatigue causes frequent problems for me; item 6: my fatigue prevents sustained physical functioning; item 7: fatigue interferes with carrying out certain duties and responsibilities; item 9: fatigue interferes with my work, family, or social life; T1: baseline assessment; T1: after 3 months of diet assessment.

	T0	T1	*p*-Value
Item 1	6.263 ± 1.310	4.947 ± 2.052	<0.001
Item 2	5.289 ± 1.486	4.184 ± 1.853	<0.001
Item 3	4.697 ± 2.142	3.763 ± 2.103	<0.001
Item 4	5.408 ± 1.798	4.263 ± 2.043	<0.001
Item 5	4.526 ± 2.043	3.592 ± 2.054	<0.001
Item 6	4.987 ± 1.851	3.921 ± 2.140	<0.001
Item 7	4.842 ± 1.960	3.500 ± 2.030	<0.001
Item 8	4.461 ± 2.484	3.632 ± 2.214	<0.001
Item 9	4.316 ± 2.276	3.395 ± 2.123	0.003
Total score	4.977 ± 1.644	3.911 ± 1.779	<0.001
Total score: LGID	5.030 ± 1.614	4.051 ± 1.740	<0.001
Total score: 2:1	5.016 ± 1.794	3.958 ± 1.945	0.034
Total score: VLCKD	4.762 ± 1.604	3.429 ± 1.678	0.006
Total score: chronic migraine	5.038 ± 1.643	3.944 ± 1.729	<0.001
Total score: high-frequency episodic migraine	4.892 ± 1.669	3.856 ± 1.874	0.002

**Table 5:** The correlation between the mean FSS reduction and the mean reduction in MIDAS, HIT-6, migraine frequency, migraine intensity, FM, and BMI. The reduction in the FSS correlates with the reduction in the HIT-6 and MIDAS scores. The correlation was performed with Spearman’s test; significance was set at a *p*-value of <0.05. Legend = FM: fat mass; FFM: fat-free mass; BMI: body mass index; MIDAS: Migraine Disability Assessment Test; HIT-6: Headache Impact Test 6; NRS: Numeric Pain Rating Scale.

	Mean FSS Reduction
Mean MIDAS reduction	r = 0.361 *p* = 0.001
Mean HIT-6 reduction	r = 0.344 *p* = 0.002
Mean frequency (days/month) reduction	r = 0.127 *p* = 0.296
Mean intensity (NRS) reduction	r = −0.196 *p* = 0.090
Mean FM reduction	r = 0.016 *p* = 0.888
Mean BMI reduction	r = −0.053 *p* = 0.652

## Data Availability

Not applibacle.

## Abbreviations

FSS: fatigue severity scale; KD: ketogenic diet; LGID: low-glycemic-index diet; VLCKD: very-low-calorie ketogenic diet; LCKD: low-calorie ketogenic diet; MAD: Modified Atkins Diet; VLCnKD: very-low-calorie, not-ketogenic diet; LCD: low-carbohydrate diet; FM: fat mass; FFM: fat-free mass; BMI: body mass index; MIDAS: Migraine Disability Assessment Test; BIA: Bioelectrical Impedance Analysis; HIT-6: Headache Impact Test 6; SD: standard deviation; NRS: Numeric Pain Rating Scale; MOH: medication overuse headache; VAS: visual analog scale; MSQ: Migraine-Specific Quality-of-Life Questionnaire (MSQ); PHQ-9: Patient Health Questionnaire-9; ESS: Epworth Sleepiness Scale; ISI: Insomnia Severity Index; GAD-7: Generalized Anxiety Disorder-7; ASC-12: 12-Item Allodynia Symptom Checklist.
